# *O*-Glycosylated Oncofetal Fibronectin Expression in Lesions of Human Cutaneous Leishmaniasis: A Novel Finding in Infectious Diseases

**DOI:** 10.3390/biology15171532

**Published:** 2026-09-03

**Authors:** Marcos André Rodrigues da Costa Santos, Diego Folena Custodio, Carolina Bruno Rufino, Jhenifer Santos dos Reis, Ellen Victoria dos Santos Tavares Pimentel, Gabrielly Silva Santos, Elias Barbosa da Silva-Junior, Joana D’Arc da Silva Trindade, José Osvaldo Previato, Lucia Mendonça-Previato, Alexandre Morrot, Leonardo Marques da Fonseca, Debora Decote-Ricardo, Celio Geraldo Freire-de-Lima, Raphael do Carmo Valente, Cintia Xavier Mello, Claude Pirmez, Marcia Pereira de Oliveira Duarte, Leonardo Freire-de-Lima

**Affiliations:** 1 Programa de Pós-Graduação em Ciências Biológicas (Biofísica), Instituto de Biofísica Carlos Chagas Filho, Universidade Federal do Rio de Janeiro, Rio de Janeiro 21941-902, RJ, Brazil; msantos@biof.ufrj.br (M.A.R.d.C.S.); diegorj2004@gmail.com (D.F.C.); carolinabr@ymail.com (C.B.R.); jheniferreis@biof.ufrj.br (J.S.d.R.); victoriapimentelufrj@gmail.com (E.V.d.S.T.P.); gsilva811@gmail.com (G.S.S.); eliasbarbosa@biof.ufrj.br (E.B.d.S.-J.); joanadarc@biof.ufrj.br (J.D.d.S.T.); previato@biof.ufrj.br (J.O.P.); luciamp@biof.ufrj.br (L.M.-P.); lfonseca@biof.ufrj.br (L.M.d.F.); celio@biof.ufrj.br (C.G.F.-d.-L.); raphael.valente@xerem.ufrj.br (R.d.C.V.); 2 Programa de Pós-Graduação em Ciências Morfológicas, Instituto de Ciências Biomédicas, Universidade Federal do Rio de Janeiro, Rio de Janeiro 21941-902, RJ, Brazil; 3 Laboratório de Imunoparasitologia, Instituto Oswaldo Cruz, Rio de Janeiro 21040-361, RJ, Brazil; alexandre.morrot@ioc.fiocruz.br; 4 Faculdade de Medicina, Universidade Federal do Rio de Janeiro, Rio de Janeiro 21941-902, RJ, Brazil; 5 Curso de Medicina, Universidade Castelo Branco, Rio de Janeiro 21710-255, RJ, Brazil; 6 Departamento de Microbiologia e Imunologia Veterinária, Instituto de Veterinária, Universidade Federal Rural do Rio de Janeiro, Seropédica 23897-000, RJ, Brazil; decotericardo96@gmail.com; 7 Campus Duque de Caxias Professor Geraldo Cidade, Universidade Federal do Rio de Janeiro, Duque de Caxias 25240-005, RJ, Brazil; 8 Laboratório Interdisciplinar de Pesquisas Médicas, Instituto Oswaldo Cruz, Rio de Janeiro 21040-361, RJ, Brazil; cintia.mello@ioc.fiocruz.br (C.X.M.); claude.pirmez@fiocruz.br (C.P.)

**Keywords:** *O*-glycosylated oncofetal fibronectin, extracellular matrix, leishmaniasis, glycosylation, glycoprotein

## Abstract

Cutaneous leishmaniasis (CL) is a chronic infectious disease characterized by extensive tissue destruction and profound extracellular matrix (ECM) remodeling. This study provides the first evidence of *O*-glycosylated oncofetal fibronectin (onf-FN) expression in human CL lesions. Immunohistochemical analysis revealed increased onf-FN expression throughout the inflammatory lesions, displaying a distribution pattern closely resembling that observed in human tumors and overlapping with total fibronectin expression. The detection of onf-FN in an infectious disease caused by protozoan parasites broadens the current understanding of ECM remodeling beyond cancer and developmental processes, suggesting that onf-FN may also participate in the host tissue response to chronic infection. Given the well-established biological functions of onf-FN in regulating cell adhesion, tissue remodeling, and repair, its expression in CL lesions raises important questions regarding its potential role in disease pathogenesis and parasite persistence. These findings identify onf-FN as a previously unrecognized component of the CL microenvironment, providing a foundation for future studies investigating its biological and clinical significance in parasitic diseases.

## Simple Summary

Cutaneous leishmaniasis is a disease caused by parasites that infect the skin. It can produce long-lasting wounds and cause considerable damage to the affected tissue. In this study, we found a particular form of a protein called fibronectin in skin lesions from people with cutaneous leishmaniasis. Fibronectin helps organize and support tissues and can also influence how cells behave during tissue damage and repair. The form identified in our study, known as oncofetal fibronectin, has previously been mainly associated with cancer and developing tissues. We found that it was widely present in areas of inflammation in leishmaniasis lesions, with a pattern similar to that seen in tumors. This is the first report of this form of fibronectin in human cutaneous leishmaniasis. Our findings suggest that some of the tissue changes occurring during this long-lasting infection may resemble those found in tumors and open new possibilities for studying how fibronectin may contribute to tissue damage, repair, and the persistence of the disease.

## 1. Introduction

Cutaneous leishmaniasis (CL) is a neglected tropical disease of skin and mucosal tissues caused by protozoan parasites from the genus *Leishmania* (Protozoa, Trypanosomatida, Trypanosomatidae) and transmitted to humans and other mammals by female infected phlebotomine sandflies. An estimated 1 million new cases occur each year, mostly in endemic areas of Latin America, the Mediterranean basin, Southwest Asia and the Middle East [1]. The disease burden mostly affects low-income populations that are subject to precarious housing and malnutrition, which impairs immune system function and disease control [2]. Most infected individuals present subclinical conditions, and parasites might persist through specific escape mechanisms [3,4]. Nevertheless, the infection can progress to CL, the most common clinical presentation, defined by the development of chronic painless ulcerations. CL lesions caused by *Leishmania (Viannia) braziliensis* can heal spontaneously, but therapy is mandatory since it speeds the healing process and reduces the risk of developing a mucosal disease or relapsing [5].

The tissue destruction in CL is a consequence of an intense and diffuse chronic inflammatory infiltrate, characterized by necrosis and a predominance of lymphocytes, macrophages (MΦ), and mast cells, frequently accompanied by a granulomatous reaction. A crucial aspect of CL is the presence of amastigotes inside MΦ, usually in the subepidermal region. The inflammatory infiltrate of CL caused by *Leishmania (V.) braziliensis* is accompanied by a low parasite load [6] and although parasite load is not a good predictor of severe forms, the parasite burden has been shown to increase with successive relapses [7].

Research on the involvement of the extracellular matrix (ECM) components in the development of CL remains limited. Nevertheless, experimental studies have demonstrated an association between increased fibronectin (FN) and TGF-β expression in susceptible mouse strains, suggesting a role for ECM remodeling during disease progression [8,9]. Accumulating evidence highlights how ECM glycoproteins mediate parasite–host interactions and influence the outcome of various infectious diseases [10,11]. Despite these advances, the glycophenotypic characterization of these glycoconjugates remains largely unexplored. In many cases, ECM molecules are described solely based on their protein backbone, with little attention given to the structural and functional implications of their glycosylation patterns. In the present study, we demonstrate for the first time the expression of an FN isoform known as *O*-glycosylated oncofetal fibronectin (onf-FN) in lesions of human CL. The onf-FN is known to carry the VTHPGY hexapeptide in the IIICS domain, in which the threonine (Thr) residue can be *O*-glycosylated by a polypeptide N-acetylgalactosaminyltransferase (pp-GalNAc-T), generating the minimal binding epitope for the FDC-6 mAb, originally described by Hakomori’s group [12,13]. Following the addition of GalNAc, other monosaccharides, such as galactose (Gal) and sialic acid (Sia), can be sequentially added by the action of additional glycosyltransferases, generating more complex *O*-glycan structures, including the T-antigen (Galβ1-3GalNAcα-O-Ser/Thr) and sialyl-T epitope (Siaα2-3Galβ1-3GalNAcα-O-Ser/Thr), both of which remain recognized by the FDC-6 mAb on the oncofetal glycoprotein [14]. Since its discovery in 1985, onf-FN has often been used as a glycobiomarker, and after 26 years, in 2011, Freire de Lima et al. [15] described increased expression of the oncofetal glycoprotein during the epithelial–to—mesenchymal transition (EMT) in human prostate epithelial cell lines treated with TGF-β, suggesting that this glycoprotein would play a role in this process, as subsequently confirmed by Ding et al. one year later [16]. Further studies have described the expression of onf-FN, as well as the potential involvement of the pp-GalNAc-T6, which is implicated in its biosynthesis [17], in the acquisition and/or maintenance of the multidrug resistance (MDR) phenotype [18,19,20]. In 1991, Lockwood et al. established FDC-6-positive FN in cervical secretions and vaginal fluid as a potential predictor of spontaneous preterm delivery [21]. Subsequent evidence demonstrated the presence of onf-FN in the plasma of non-pregnant individuals, with concentrations reaching up to 12 μg/mL [22]. Although this oncofetal glycoprotein has been extensively studied in various types of cancer, its expression has been reported in several non-malignant conditions, suggesting that it may play broader roles than previously recognized [23,24,25,26,27,28,29]. Collectively, these observations suggest that glycosylation represents an important yet understudied layer of ECM regulation. A deeper understanding of the glycan signatures associated with matrix glycoproteins may reveal novel aspects of host–pathogen interactions and provide valuable insights into the molecular mechanisms governing infectious disease pathogenesis.

## 2. Materials and Methods

### 2.1. Patients

Six patients presenting with active CL and residing in endemic areas of Rio de Janeiro State, Brazil, were included in this study. The diagnosis of CL was established based on clinical, epidemiological, and laboratory findings. Incisional biopsies were obtained from the edge of active lesions under local anesthesia. Each biopsy specimen was divided into two fragments. One fragment was fixed in 10% buffered formalin, embedded in paraffin, and stained with hematoxylin and eosin (H&E) for histopathological analysis, as previously described [5]. The second fragment was subjected to DNA extraction, followed by molecular detection of *Leishmania* by kDNA-PCR, as previously described [30]. All suspected patients were confirmed to be positive for CL. The definitive diagnosis of CL was established by the presence of amastigotes upon microscopic examination and/or by the detection of the 120 bp minicircle fragment on 8% polyacrylamide gel following kDNA-PCR amplification. As negative controls, three samples of normal skin obtained from adult subjects undergoing esthetic surgery were included. Tissue fragments from patients with basal cell carcinoma (BCC) were used as positive controls. Clinical data and tissue fragments from all CL patients were collected before the initiation of pentavalent antimony treatment. CL patients with comorbidities such as diabetes, hypertension, or co-infections were excluded from the study. Histopathological and molecular analyses were performed as described below.

### 2.2. Histopathological Analyses

Skin biopsy specimens collected from cutaneous lesions were immediately immersed in 10% neutral buffered formalin (NBF) (Sigma-Aldrich, St. Louis, MO, USA) at a volume of at least 20 times that of the tissue and fixed at room temperature for 24 to 48 h. Following fixation, the tissues were dehydrated through a graded series of ethanol solutions (70%, 80%, 95%, and 100%), cleared in xylene (Sigma-Aldrich), and embedded in premium-grade paraffin wax (Leica Biosystems, Wetzlar, Germany). Serial sections of 4 µm thickness were cut using a rotary microtome (model RM2235, Leica Biosystems, Wetzlar, Germany), floated onto a warm water bath at 42 °C, transferred onto glass microscope slides, and dried overnight at room temperature. Before staining, paraffin-embedded sections were completely dried at 58 °C for 20–30 min to ensure optimal tissue adhesion. Then, tissue sections were deparaffinized in two changes of xylene for 5 min each and rehydrated through a decreasing graduated series of ethanol (three changes in 100% ethanol, followed by one change each in 95%, 80%, and 70% ethanol) for 3 min per step, followed by a final rinse in distilled water. Histopathological evaluation was performed using routine H&E staining. Briefly, rehydrated sections were stained with Hematoxylin 560 MX solution (Leica Biosystems) for 2 min to target nucleic components. The slides were rinsed in running tap water for 10 min, and the cytoplasmic structures were counterstained by immersion in Eosin 515 Phloxine solution (Sigma-Aldrich) for 1 min. The sections were rapidly dehydrated through an increasing graded series of ethanol (95% and twice in 100%) for 1 min each, cleared in two changes of xylene for 5 min, and permanently mounted using CV Mount (Leica Biosystems) under glass coverslips. The stained tissue sections were examined under a brightfield light microscope (Axio Imager M2 microscope (Carl Zeiss Microscopy GmbH, Jena, Germany)) equipped with a digital camera system. Tissue architecture, inflammatory infiltrates, granuloma formation, and the presence of intracellular structures (such as *Leishmania* amastigotes) were evaluated qualitatively by an experienced pathologist at 400× and 1000× magnifications.

### 2.3. Immunohistochemical Detection of Total and O-Glycosylated Onf-FN

IHC staining was performed on 4-µm-thick formalin-fixed, paraffin-embedded skin biopsy tissue sections to evaluate the expression patterns and distribution of total and onf-FN. Sections were completely dried at 58 °C for 20–30 min to ensure optimal tissue adhesion. Then, sections were deparaffinized in two changes of xylene for 5 min each and rehydrated through a decreasing graded series of ethanol (three changes in 100% ethanol, followed by one change each in 95%, 80%, and 70% ethanol) for 3 min per step, followed by rinsing in distilled water. Endogenous peroxidase activity was blocked by incubating the slides in a 0.6% hydrogen peroxide (H_2_O_2_) solution in methanol for 15 min at room temperature in the dark. For antigen retrieval, sections were immersed in a 10 mM sodium citrate buffer (pH 6.0) and heated in a water bath at 95–98 °C for 20 min, followed by a 20-min cooling period at room temperature. To prevent non-specific protein binding, sections were incubated with a 2% BSA blocking solution in Tris-HCl, pH 7.4 for 30 min at room temperature. The tissue sections were incubated overnight at 4 °C in a humidified chamber with primary antibodies against total FN (EP5; Santa Cruz Biotechnology, Dallas, TX, USA; 1:40 dilution) or onf-FN (FDC-6; 1:40 dilution), the latter kindly provided by Dr. Sen-Itiroh Hakomori (Seattle, WA, USA). Both antibodies were diluted in PBS containing 1% BSA. FDC-6 was purified from antibody-enriched hybridoma culture supernatant using Protein G resin (GenScript, Piscataway, NJ, USA), as previously described [16]. Following purification, aliquots at a concentration of 500 µg/mL were stored at −20 °C until use in the biological assays. Negative controls were prepared by replacing primary antibodies with PBS alone. Following three 5 min washes with Tris-HCl, immunoreactivity was detected using the Novolink™ Polymer Detection System (Leica Biosystems) according to the manufacturer’s instructions. Immunostaining was visualized with 3,3′-diaminobenzidine (DAB) as the chromogen, and the sections were counterstained with Hematoxylin 560 MX solution for 3 min (Leica Biosystems). Sections were dehydrated through an increasing graded series of ethanol, cleared in xylene, and permanently mounted under glass coverslips using CV Mount (Leica Biosystems). For quantitative image analysis, ten randomly selected microscopic fields were acquired from each tissue section under identical imaging conditions. DAB staining intensity was quantified via digital densitometric analysis using Image-Pro Plus 7.0 software (Media Cybernetics, Rockville, MD, USA). Digitally captured images from the different tissues included in the study were analyzed by selecting the specific tissue boundaries (Region of Interest, ROI) to avoid background interference. Following color deconvolution for DAB signal separation, the mean gray value was measured and converted into Optical Density (OD). The final staining intensity was calculated to compare protein expression levels. All sections were examined using an Axio Imager M2 microscope (Carl Zeiss, Jena, Germany) by two experienced observers.

### 2.4. PCR Analysis

DNA was extracted from cutaneous leishmaniasis lesion biopsy specimens using the DNeasy Blood & Tissue Kit (Qiagen, Hilden, Germany) according to the manufacturer’s instructions. Tissue lysis was performed by incubating the samples with 180 µL of Buffer ATL and 20 µL of Proteinase K (Qiagen) at 56 °C in a Digital Block Heater (Thermo Fisher Scientific, Waltham, MA, USA) until complete digestion. Following lysis, 200 µL of Buffer AL and 200 µL of absolute ethanol (Sigma-Aldrich) were added to the lysate. The mixture was loaded onto a DNeasy Mini Spin Column and washed sequentially with 500 µL of Buffer AW1 and 500 µL of Buffer AW2. The column was dried by centrifugation at 20,000× *g* for 3 min. Total DNA was eluted in 20 µL of Buffer AE after a 5-min incubation at room temperature. The concentration and purity of the extracted DNA were determined using a NanoDrop 2000 Spectrophotometer (Thermo Fisher Scientific). Only aliquots with an *A*_260_/*A*_280_ purity ratio between 1.8 and 2.0 were stored at −20 °C and utilized in subsequent PCR assays. DNA samples were initially screened by PCR amplification of the endogenous human β-actin gene to assess DNA integrity and confirm the absence of PCR inhibitors. Only samples showing the expected 408-bp product were subsequently analyzed by kDNA PCR for *Leishmania* detection, as previously described [30]. Total DNA extracted from promastigote cultures of the *Leishmania* (*V.*) *braziliensis* reference strain MHOM/BR/75/2903 was used as the positive control. Amplification of the *Leishmania* kinetoplast DNA (kDNA) minicircles was performed using the primers HM1: 5′-(CCG CCC CTA TTT TAC ACC AAC CCC)-3′; HM2: 5′-(GGG GAG GGG CGT TCT GCG AA)-3′; HM3: 5′-(GGC CCA CTA TAT TAC ACC AAC CCC)-3′. PCR assays were carried out in a final volume of 25 µL containing 0.2 µL of Taq Platinum DNA polymerase (5 µ/L), 2.5 µL of buffer (10×), 0.25 µL of each primer (HM1/HM2 12.5 ng and HM3 50 ng), 0.75 µL of MgCl2 (50 mM), 0.5 µL of dNTP (10 mM), 18.3 µL of Nuclease-Free Water (Thermo Fisher Scientific), and 2.0 µL of the DNA template. The reaction was performed in a 96-Well Thermal Cycler Gene Amp PCR System 9700 (Applied Biosystems, Foster City, CA, USA) under the following conditions: initial denaturation at 94 °C for 1 min; 35 cycles of denaturation at 94 °C for 30 s, annealing at 54 °C for 30 s, and extension at 72 °C for 45 s; followed by a final extension step at 72 °C for 5 min. The PCR amplification product (120 bp) was resolved by electrophoresis on 8% polyacrylamide gel prepared in 1× TBE buffer followed by silver staining Plus Kit for DNA visualization (Bio-Rad Laboratories, Hercules, CA, USA). Samples (10 µL) were loaded directly into the wells, alongside a DNA Ladder (Thermo Scientific) as a molecular weight standard. Electrophoresis was conducted at 60 V for 90 min in a Sub-Cell GT Cell system (Bio-Rad Laboratories).

### 2.5. Statistical Analysis

Data were expressed as means ± standard deviations (SDs). Statistical analyses and graphical representations were performed using GraphPad Prism software (version 6.0, GraphPad Software, San Diego, CA, USA). Data distribution was evaluated, and differences between groups were assessed for statistical significance (*p* ≤ 0.05) using the non-parametric Mann–Whitney, Kruskal–Wallis and Wilcoxon tests.

## 3. Results

### 3.1. Clinical Cohort and Diagnostic Confirmation

A total of 12 individuals were evaluated in this study, including five males and seven females, with ages ranging from 19 to 51 years. Their demographic characteristics, clinical diagnoses, disease duration, and laboratory diagnostic findings are summarized in Table 1. Six patients (Patients 1–6) had a clinical diagnosis of CL, comprising three males and three females, with ages ranging from 19 to 51 years and disease duration ranging from 1 to 3 months. All six CL cases were laboratory-confirmed by at least one diagnostic modality. Patients 1–4, comprising two males and two females aged 32–51 years, were positive for amastigotes by imprint/histopathological analysis, with disease duration of 2–3 months, whereas molecular testing by polymerase chain reaction (PCR) was not determined (ND) for this subgroup. Patients 5 and 6, one female and one male aged 19 and 47 years, respectively, had disease durations of 1 and 2 months and were laboratory-confirmed positive exclusively by PCR, while their imprint/histopathology status remained ND. Patients 7–9 were diagnosed with BCC and comprised two males and one female, aged 36–45 years; imprint/histopathological analysis was negative in all three cases, whereas kDNA-PCR was ND. Finally, Patients 10–12 had normal skin and comprised three females aged 36–48 years, serving as negative controls. Disease duration was not applicable for these individuals, and neither imprint/histopathological analysis nor kDNA-PCR was performed.

### 3.2. Molecular and Histopathological Characterization of CL Lesions

**Figure 1:**
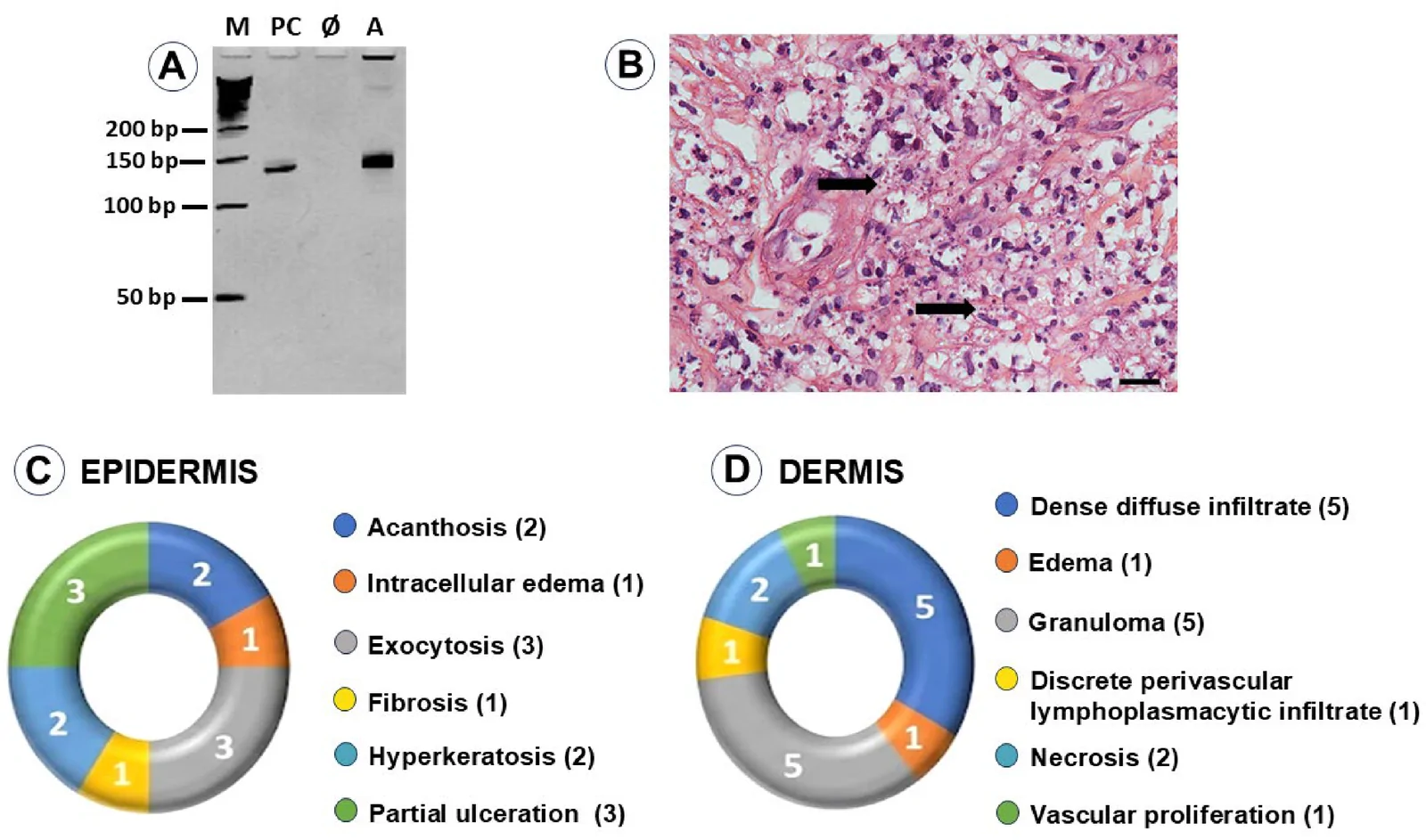
Molecular, Histopathological, and Microenvironmental Characterization of Cutaneous Leishmaniasis Lesions. (**A**) Representative polyacrylamide gel electrophoresis of polymerase chain reaction products confirming the presence of *Leishmania* kinetoplast DNA (kDNA). A distinct diagnostic band at 120 bp is visible relative to the molecular weight marker (M). Lane PC: Positive Control (DNA extracted from *Leishmania (V.) braziliensis* promastigote cultures); Lane ∅: Negative Control (nuclease-free water template); Lane A: Confirmed clinical sample. (**B**) Histopathological micrograph of a skin biopsy section stained with hematoxylin and eosin (H&E). The section reveals an intense inflammatory cellular infiltrate within the dermis. Black arrows indicate intracellular *Leishmania* amastigotes clustered within the MΦ cytoplasm. (Scale bar = 10 µm). (**C**) Quantitative distribution and frequency of histopathological alterations observed within the epidermal compartment of the evaluated CL lesions. (**D**) Quantitative distribution and frequency of structural, inflammatory, and vascular alterations quantified within the dermal compartment of the evaluated CL lesions. Numbers within the donut charts represent the total number of cases (*n*) exhibiting each feature.

To characterize the infection and tissue microenvironment, molecular and histopathological analyses were performed on the confirmed CL cases (Figure 1). Diagnostic PCR amplification confirmed the presence of *Leishmania* kDNA (120 bp), yielding a distinct target band visible relative to the molecular weight marker (M). The positive control (PC) consisted of DNA extracted from promastigote cultures of *Leishmania (V.) braziliensis*, while the negative control (Ø), which consisted of the PCR master mix containing nuclease-free water instead of a DNA template, remained clear (Figure 1A). Histopathological examination of the cutaneous lesions stained with hematoxylin and eosin (H&E) revealed an intense cellular response with intracellular *Leishmania* amastigotes within mΦ (Figure 1B, arrows). Regarding the histopathological alterations in the epidermis and dermis, quantitative assessment of the structural tissue changes across the evaluated biopsy samples demonstrated widespread inflammatory and reactive alterations (Figure 1C,D). In the epidermis (Figure 1C), the most prevalent architectural alterations were partial ulceration and exocytosis, each observed in three cases, followed by acanthosis (*n* = 2) and hyperkeratosis (*n* = 2). Intracellular edema and fibrosis were less frequent; each identified in a single case (*n* = 1). In the dermal compartment (Figure 1D), marked inflammatory features were observed. Both a dense diffuse infiltrate and granuloma formation were the most dominant findings, each characterized in five cases. Necrosis was identified in two cases, while tissue edema, vascular proliferation, and discrete perivascular lymphoplasmacytic infiltrate were each detected in one case.

### 3.3. Immunohistochemical Expression of Total and Oncofetal Fibronectin

**Figure 2:**
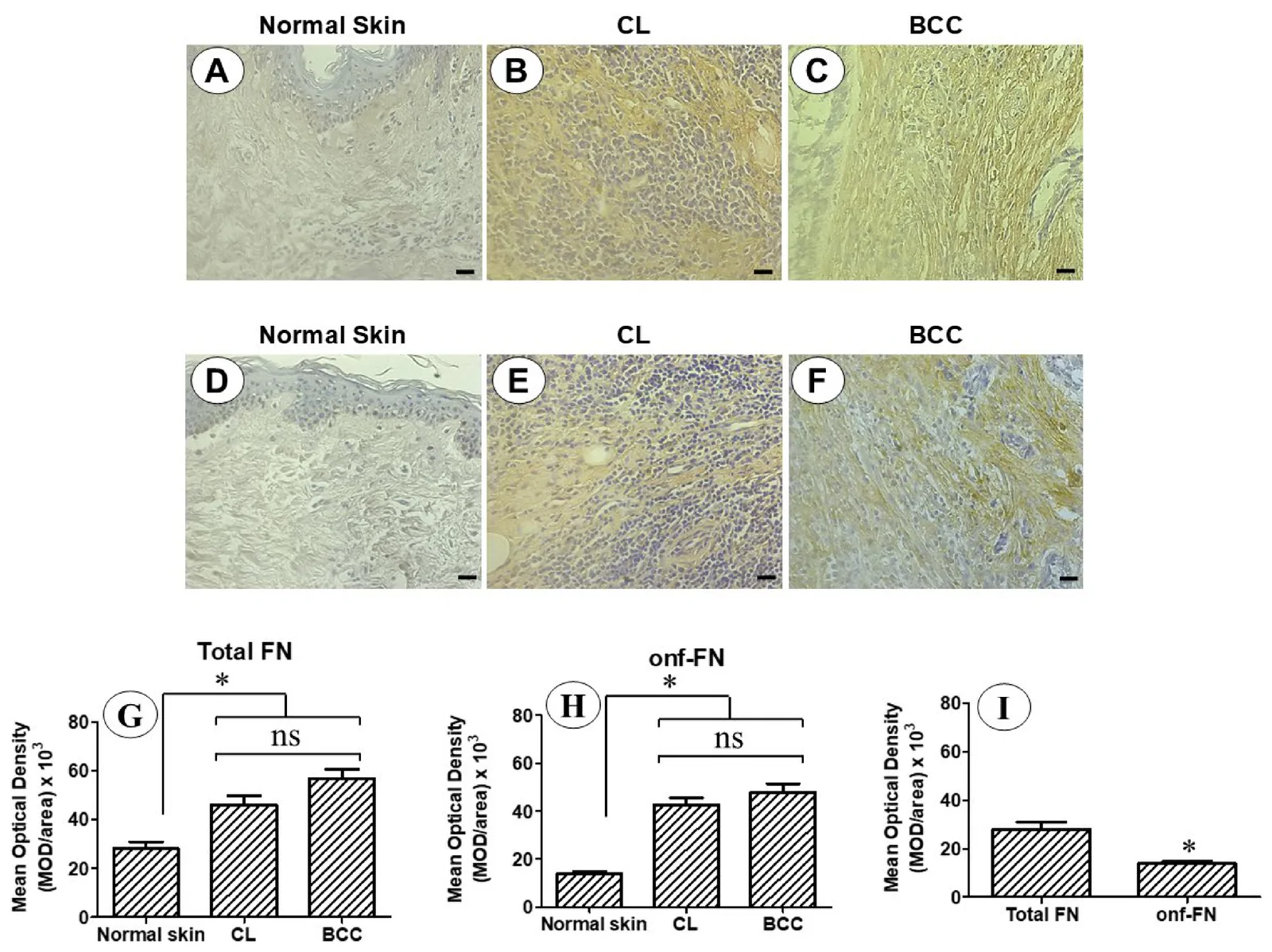
Immunohistochemical expression and quantitative analysis of total FN and onf-FN in normal skin, cutaneous leishmaniasis (CL), and basal cell carcinoma (BCC). Representative photomicrographs showing immunoreactivity for total FN (EP5; (**A**–**C**)) and onf-FN (FDC-6; (**D**–**F**)) in normal skin (**A**,**D**), CL (**B**,**E**), and BCC (**C**,**F**). Nuclei were counterstained with hematoxylin. Scale bars = 20 μm. Bar graphs represent the mean optical density (MOD/area × 10^3^) for total FN (**G**) and onf-FN (**H**). (**I**) Comparison of total FN and onf-FN expression in healthy human skin. Quantitative values were replotted from panels G and H and combined into a single graph for comparison only. Mean optical density (MOD/area × 10^3^) analysis demonstrates that both FN isoforms are constitutively expressed in healthy skin, with onf-FN exhibiting significantly lower levels than total FN. Data are expressed as means ± SDs. * *p* ≤ 0.05; ns = non-significant.

To evaluate the deposition and distribution of FN isoforms in tissue microenvironments, IHC analysis was performed for total FN (EP5) and onf-FN (FDC-6) across normal skin, CL, and BCC samples (Figure 2). Regarding total FN, normal skin exhibited weak and restricted immunoreactivity in the dermis (Figure 2A). In contrast, both CL and BCC tissues showed a marked increase in EP5 expression, characterized by intense and diffuse stromal staining (Figure 2B,C). Quantitative analysis of the mean optical density (MOD) confirmed a significant increase in total FN deposition in BCC compared to normal skin (*p* ≤ 0.05; Figure 2G), whereas no statistically significant difference was observed between CL and BCC groups. A similar pattern was observed for onf-FN. FDC-6 staining was detectable in normal skin, although at low levels, particularly in the subepidermal basement membrane zone and superficial dermis (Figure 2D). However, onf-FN expression was strongly upregulated in the stroma of both CL and BCC lesions, displaying a fibrillar pattern surrounding the inflammatory infiltrate and tumor nests, respectively (Figure 2E,F). Morphometric quantification revealed that onf-FN levels were significantly higher in BCC lesions than in normal skin (*p* ≤ 0.05; Figure 2H), with no significant difference (ns) detected between the CL and BCC groups. To further characterize FN expression under physiological conditions, we extracted the data from Figure 2G,H to compare EP5 and FDC6 staining exclusively in healthy skin samples. As shown in Figure 2I, pan-fibronectin (EP5) staining was significantly higher than FDC6 immunoreactivity. Nevertheless, the FDC6-reactive glycoform was consistently detected in healthy samples, suggesting that the *O*-glycosylated onf-FN isoform may be expressed at low basal levels in normal skin. In all samples, negative controls were prepared by replacing the primary antibodies with PBS alone. These findings suggest that this glycoform may not be strictly restricted to cancer, providing support for a potential physiological role in some normal tissues. Furthermore, its baseline presence in healthy skin points to the possibility that onf-FN could contribute to early interactions between *Leishmania* parasites and resident phagocytes during the initial stages of infection. Although this hypothesis remains to be experimentally validated, it opens new avenues for investigating the role of carbohydrate-mediated interactions during protozoan parasite infection and may expand our understanding of the physiological functions of this glycoform beyond cancer.

## 4. Discussion

This study provides the first evidence of the expression of *O*-glycosylated onf-FN in the tissue microenvironment of human CL lesions. Known to be expressed in fetal tissues and tumor cells, as observed in the BCC samples used here as a positive control, the de novo expression of onf-FN in an infectious-inflammatory context introduces a novel dimension to the understanding of ECM remodeling during chronic *Leishmania* infection. The histopathological profile of our clinical cohort showed intense dermal inflammatory responses, marked by dense diffuse infiltrates, granuloma formation, and the presence of amastigotes within MΦ. Quantitative analysis demonstrated that the expression levels of total FN and onf-FN in CL lesions were significantly upregulated compared to normal skin, reaching levels similar to those observed in BCC. In the context of *Leishmania* infection, the overproduction of FN has complex biological implications. FN is known to act as a scaffold for cell adhesion, migration, and tissue repair [31]. However, several pathogens, including *Leishmania* spp., exploit host ECM proteins to facilitate attachment, internalization, and immune evasion [32]. Previous studies have demonstrated that host-derived FN promotes the attachment of both promastigote and amastigote forms of *Leishmania* to mononuclear phagocytes, facilitating their entry into host cells [33]. Key parasite surface proteins are described for their ability to mediate this interaction. For instance, the major surface metalloprotease gp63 has been described as a FN-like molecule that can interact with host cell integrin receptors to promote parasite internalization without triggering a strong respiratory burst [32,34]. Furthermore, once inside the host dermis, *Leishmania* parasites actively alter the composition and physical properties of the ECM [35]. It has been well described that *Leishmania* promastigotes and amastigotes secrete proteases. Examples include gp63 and cysteine proteases, which can proteolytically degrade host FN [32,36]. Rather than being a simple byproduct of infection, this FN degradation plays a strategic role in pathogenesis by actively modulating MΦ activation and impairing host cell migration. Specifically, while intact FN generally stimulates MΦ to promote an inflammatory phenotype and trigger the release of reactive oxygen species (ROS) or TNF-α [37], *Leishmania*-degraded FN fragments suppress the production of ROS and downregulate key pro-inflammatory cytokines, thereby dampening the host’s microbicidal response to favour parasite survival and persistence in the infected host [36]. At the same time, this proteolytic breakdown alters host cellular recruitment and localization. Although cell migration is essential for effective immune surveillance, *Leishmania* infection has been shown to alter the dynamics of MΦ and dendritic cell migration in three-dimensional environments, anchoring or misdirecting host cells [38]. Studies indicate that *Leishmania* modulates β1-integrin activation, specifically VLA-4, on monocytes, which impairs their normal spreading and adhesion kinetics on FN-coated surfaces, potentially hindering the coordination of an effective localized immune response and promoting parasite persistence and dissemination [32,39]. The prominent role of TGF-β as a major driver of FN deposition and host–cell invasion by pathogens is not restricted to *Leishmania* infection but represents a conserved strategy among other kinetoplastid parasites. For example, in non-immune cells such as cardiomyocytes, treatment with TGF-β significantly upregulates ECM components, notably inducing a robust increase in FN expression which directly facilitates the adhesion and internalization of *Trypanosoma cruzi*. Since our previous study has shown that treating epithelial cells with TGF-β significantly increases the expression of onf-FN [15], it is plausible to speculate that the FN expressed by cardiomyocytes treated with TGF-β is the *O*-glycosylated oncofetal isoform. However, this hypothesis needs to be experimentally confirmed. Interestingly, after the parasite successfully penetrates the cell, FN expression is subsequently downregulated [40]. This phenomenon may be a finely tuned evolutionary mechanism designed to prevent superinfection. By decreasing FN levels post-invasion, the host cell or the parasite limits the entry of *T. cruzi* trypomastigotes, thereby preventing excessive intracellular overcrowding. Without this regulatory mechanism, a massive accumulation of amastigotes would lead to early, premature rupture of the host cell before the parasites could complete their intracellular cycle and differentiate into the infective trypomastigote form to propagate the infection to neighboring tissues. The identification of *O*-glycosylated onf-FN in CL lesions adds a critical layer of post-translational regulation to this host–pathogen dynamic. The *O*-glycosylation of the hexapeptide VTHPGY within the IIICS domain of FN, which defines the onf-FN glycoform, is known to alter the conformation of the glycoprotein, rendering it more flexible and potentially altering its susceptibility to proteolysis. Crucially, it induces conformational changes in the glycoprotein, exposing binding sites for new epitopes (such as the FDC-6 mAb binding site) while simultaneously masking or concealing other existing ligand-binding sites [41]. This conformational flexibility likely influences how the molecule interacts with both host cell integrins and parasite-specific surface receptors. Therefore, the biological significance of this oncofetal glycoprotein, initially proposed and investigated primarily as a fetal and tumor biomarker, needs to be thoroughly explored within the context of infectious diseases. To better understand the direct and distinct roles of the *O*-glycosylated (oncofetal) and non-glycosylated (normal) isoforms of FN during *Leishmania* infection, as well as infections by other protozoan parasites, future experimental strategies must rely on the physical separation of these molecules. The dynamic interaction with FN motifs is a widely shared mechanism across diverse parasitic phyla; for instance, *Plasmodium falciparum*-parasitized erythrocytes have been shown to bind to the RGD motif of immobilized FN on endothelial cells and platelets via a band 3-related adhesin through its HPLQKTY peptidic sequence [42].

Importantly, the detection of the FDC-6-reactive FN glycoform in healthy skin, albeit at significantly lower levels than total FN, raises the intriguing possibility that this glycoform is not strictly restricted to pathological tissues. However, given the limited sample size in this study, these observations must be interpreted with caution and warrant further validation. Nevertheless, this finding aligns with our previous study [15], in which TGF-β-induced EMT in nonmalignant human epithelial cells markedly upregulated onf-FN expression, while detectable levels were also observed under basal conditions in both cell lysates and supernatants. This basal presence presumably suggests that onf-FN could be constitutively produced and secreted at low physiological levels. Once in the extracellular milieu, it is plausible to speculate that this glycoprotein might participate in paracrine cell-matrix signaling and local microenvironment regulation. Together with its preliminary detection in healthy skin, these findings suggest an emerging concept: onf-FN might represent a physiological glycoform present in some healthy tissues at baseline levels, markedly amplified in disease states, rather than a strictly disease-specific marker. Furthermore, its baseline presence in healthy tissues hints at a hypothetical role in physiological cell-matrix crosstalk and early host-pathogen interactions. While these speculative hypotheses remain to be rigorously tested in larger cohorts and experimental models, both this glycoform and the glycosyltransferases involved in its biosynthesis deserve further investigation. Given that conformational shifts can mask or expose such vital sequences as the RGD loop, the utilization of immunoaffinity chromatography columns to purify both FN isoforms, such as the methodology described in our previous work [16] using monoclonal antibodies to selectively isolate onf-FN and normal-FN, will be an invaluable tool to dissect the functional host–pathogen interactions mediated by these distinct ECM glycoforms in protozoan infections.

## 5. Conclusions

In conclusion, this study demonstrates for the first time the prominent expression and stromal deposition of *O*-glycosylated onf-FN in human CL lesions. Its quantitative expression in CL is highly upregulated compared to normal skin, mirroring the pattern found in epithelial malignancies like BCC and other cancer types. These findings expand the biological significance of onf-FN beyond oncology and developmental biology, establishing it as a novel component of the tissue response to chronic infectious diseases. Given the pivotal role of FN and its degradation products in mediating *Leishmania* host–cell attachment, survival, and immune modulation, the characterization of O-glycosylated onf-FN deposition in CL lesions opens new avenues for investigating tissue remodeling, chronicity, and parasite persistence in tegumentary leishmaniasis.

## Figures and Tables

**Table 1:** Clinical diagnoses and laboratory findings of the evaluated patients.

Patient ID	Age	Gender	Diagnosis	Disease Duration (Months)	Imprint/Histopathology (Amastigotes)	kDNA-PCR
1	36	M	CL	2	Positive	ND
2	41	M	CL	3	Positive	ND
3	51	F	CL	3	Positive	ND
4	32	F	CL	2	Positive	ND
5	19	F	CL	1	ND	Positive
6	47	M	CL	2	ND	Positive
7	45	M	BCC	ND	Negative	ND
8	38	F	BCC	ND	Negative	ND
9	36	M	BCC	ND	Negative	ND
10	36	F	Normal skin	N/A	ND	ND
11	41	F	Normal skin	N/A	ND	ND
12	48	F	Normal skin	N/A	ND	ND

Abbreviations: M, male; F, female; CL, cutaneous leishmaniasis; BCC, basal cell carcinoma; ND, not determined; N/A, not applicable; kDNA-PCR, kinetoplast DNA-polymerase chain reaction.

## Data Availability

Data are contained within the article. Further inquiries can be directed to the corresponding authors.
